# Supplementary material for: First Report of Anopheles annularis s.l., An. maculatus s.s., and An. culicifacies s.l. as Malaria Vectors and a New Occurrence Record for An. pseudowillmori and An. sawadwongporni in Alipurduar District Villages, West Bengal, India
Source: Microorganisms. 2024 Jan 3;12(1):95. doi: 10.3390/microorganisms12010095 (PMC10818895; doi:10.3390/microorganisms12010095)
Supplement: Supplementary file 1 [file microorganisms-12-00095-s001.zip › microorganisms-2699629-supplementary.pdf]

Supplementary materials:

Table S1: Block wise malaria data of Alipurduar district from 2017 to 2022

Year	Block/ District Total	Population	ABER (%)	Total malaria cases	API
2017	**Alipurduar-I**	220230	25.99	2	0.009
	**Alipurduar-II**	232544	44.18	71	0.305
	**Kumargram**	209946	23.417	242	1.153
	**Falakata**	304513	30.972	17	0.056
	**Kalchini**	295514	17.371	664	2.247
	**Madarihat**	210712	20.0001	319	1.514
	**District Total**	1473459	33.92	1315	0.892
2018	**Alipurduar-I**	236392	29.04	0	0.000
	**Alipurduar-II**	243714	58.79	37	0.152
	**Kumargram**	220394	27.1	47	0.213
	**Falakata**	312719	36.87	9	0.029
	**Kalchini**	297459	18.66	14	0.047
	**Madarihat**	215411	34.28	29	0.135
	**District Total**	1526089	33.66	136	0.089
2019	**Alipurduar-I**	236392	29.47	3	0.013
	**Alipurduar-II**	243714	54.88	14	0.057
	**Kumargram**	220394	40.6	328	1.488
	**Falakata**	312719	40.26	8	0.026
	**Kalchini**	297459	23.68	30	0.101
	**Madarihat**	215411	36.46	52	0.241
	**APD Municipality/ DH**	66116	16.18	22	0.333
	**District Total**	1592205	36.338	457	0.287
2020	**Alipurduar-I**	236392	10.91	2	0.008
	**Alipurduar-II**	243714	28.39	4	0.016
	**Kumargram**	220394	25.6	26	0.118
	**Falakata**	312719	17.43	2	0.006
	**Kalchini**	297459	14.49	8	0.027
	**Madarihat**	215411	18.14	2	0.009

	**APD Municipality/ DH**	66116	8.4	0	0.000
	**District Total**	1592205	18.42	44	0.028
2021	**Alipurduar-I**	228003	18.85	1	0.004
	**Alipurduar-II**	249636	26.02	6	0.024
	**Kumargram**	215917	24.12	5	0.023
	**Falakata**	312719	26.39	2	0.006
	**Kalchini**	297459	24.26	6	0.020
	**Madarihat**	218512	25.95	3	0.014
	**District Total**	1522246	23.71	23	0.015
2022	**Alipurduar-I**	245462	30.39	6	0.024
	**Alipurduar-II**	253066	36.91	15	0.059
	**Kumargram**	228850	29.68	12	0.052
	**Falakata**	324718	34.01	7	0.022
	**Kalchini**	308873	32.68	6	0.019
	**Madarihat**	223677	39.41	3	0.013
	**District Total**	1584646	32.85	49	0.031

ABER- Annual Blood Examination Rate, **API-**Annual Parasite Incidence

Table S2: The malaria cases reported from the study area in 2018-2020 and 2022.

Year	PHC	Subcentre	Village	Population	Pf cases	Pv cases	Total malaria cases	API (Malaria cases per 1000 population)
2018	Kumargram	West Chengmari	Paschim Chengmari	3398	0	0	0	0.00
			Lalchandpur	895	0	0	0	0.00
			Dhantali	940	0	1	1	1.06
			**Subcentre Total**	**5233**	**0**	**1**	**1**	**0.19**
2018	Kumargram	Turturikhanda	Turturikhanda	1552	0	3	3	1.93
			Turturi TE	2404	0	1	1	0.42
			**Subcentre Total**	**3956**	**0**	**4**	**4**	**1.01**
2018	Madhya Rangalibazna	Gopalpur T. G	Chapra Line	1402	0	0	0	0.00
			Kolkata Line	1418	0	0	0	0.00
			Jhari Line	1331	0	0	0	0.00
			Godwan Line	1210	0	0	0	0.00
			Upper Line	892	0	0	0	0.00
			**Subcentre Total**	**6253**	**0**	**0**	**0**	**0.00**
2018	Turturi	Uttar Shibkata	Uttar Shibkata	1255	0	3	3	2.39
			Srinathpur	900	0	2	2	2.22
			Maddhya Shibkata	899	0	2	2	2.22

			**Subcentre Total**	**3054**	**0**	**7**	**7**	**2.29**
2018	Turturi	Raydak TE –I	Raydak TG	4634	0	2	2	0.43
			Chipra Forest village	473	0	0	0	0.00
			Siltong Forest village	786	0	0	0	0.00
			**Subcentre Total**	**5893**	**0**	**2**	**2**	**0.34**
2019	Kumargram	West Chengmari	Paschim Chengmari	3398	0	4	4	1.18
			Lalchandpur	895	0	1	1	1.12
			Dhantali	940	0	1	1	1.06
			**Subcentre Total**	**5233**	**0**	**6**	**6**	**1.15**
2019	Kumargram	Turturikhanda	Turturikhanda	2552	0	4	4	1.57
			Turturi TE	2404	0	0	0	0.00
			**Subcentre Total**	**4956**	**0**	**4**	**4**	**0.81**
2019	Turturi	Rydak TE- I	Raydak TG	4634	2	200	202	43.59
			Chipra Forest village	473	0	0	0	0.00
			Siltong Forest village	786	0	0	0	0.00
			**Subcentre Total**	**5893**	**2**	**200**	**202**	**34.28**
2019	Kumargram	Joydebpur	Joydebpur	2620	0	5	5	1.91

			Pukharigaon	3018	0	0	0	0.00
			Amarpur	2637	0	3	3	1.14
			**Subcentre Total**	**8275**	**0**	**8**	**8**	**0.97**
2020	Kumargram	West Chengmari	Paschim Chengmari	3398	0	0	0	0.00
			Lalchandpur	895	0	0	0	0.00
			Dhantali	940	0	0	0	0.00
			**Subcentre Total**	**5233**	**0**	**0**	**0**	**0.00**
2022	Turturi	Dhowlabasti	Dhowlaj hora -I	1091	NA	NA	NA	NA
			Dhowlaj hora -II	1524	NA	NA	NA	NA
			Uttar Rampur	840	NA	NA	NA	NA
			**Subcentre Total**	**3455**	**3**	**5**	**8**	**2.32**

NA: Not available

Table S3: List of PCR primers used in the current studies

PCR	Forward (5'-3')	Reverse (5'-3')	Species	References
Maculatus group multiplex PCR	5.8F (ATCACTCGGCTCGTGGATCG) (Universal forward primer)	Mac (GACGGTCAGTCTGGTAAAGT)	*An. maculatus*	11
		PSEU (GCCCCCGGGTGTCAAACAG)	*An. pseudowillmori*	
		SAW (ACGGTCCCGCATCAGGTGC)	*An. sawadwongporni*	
		Form K (TTCATCGCTCGCCCTTACAA)	*Form K*	
		DRAV (GCCTACTTTGAGCGAGACCA)	*An. dravidicus*	
Funestus group multiplex PCR	5.8F (ATCACTCGGCTCATGGATCG) (Universal forward primer)	GGGCGCCATGTAGTTAGAGTTG	*An. minimus* A	12
		GTGGCCCCGCAATGTATG	*An. varuna*	
		AGGTTCACCCCGCTCTGG	*An. aconitus*	
		CTCCCCATAGCGCGTAAGC	*An. jeyporiensis*	
		GAAAGCACCTGAAACCTGCG	*An. pampanai*	
		GGTTGCCCACTCAATACGGGTG	*An. minimus* C	
Annularis group multiplex PCR	5.8F (TGTGAACTGCAGGACACATG) (Universal forward primer)	PHI (GCACG CCATTAT GCGACAAAC)	*An. philippinensis*	13
		NIV (CATGTA CCTCACGATACA TG TA)	*An. nivipes*	
		ANN (ACCCCTTTGCTAGCGCCCGTG)	*An. annularis*	
		PAL (GTTAAGTGAGACGATAAAGACC)	*An. pallidus*	
CO1 PCR	MBAR_F (GGATTTGGAAATTGATTAGTTCCTT)	MBAR_R (AAAAATTTTAATTCCAGTTGGAACAGC)	Universal for all mosquito species	14
Human blood meal assay	HUM1 (CGAGAGTTCTCTGGAAGAATT GA)	HUM2 (TGATAGCCTGGAAGTGACAAAAT)	*Homo sapians*	15
